# Pathology of the Teeth

**Published:** 1901-04

**Authors:** 


### Bibliography.

Pathology of the Teeth. By Carl Wedl. Second Revised Edition. Edited by Drs. Joseph Ritter von Metnitz and Gustav Ritter von Wunschheim. Vol. I. Published by Arthur Felix, of Leipzig.[1]

The first edition of Wedl's Pathology was published in 1870, and was translated into English in 1872 by W. E. Boardman, M.D., of Boston. It has during these years been held to be a standard work upon the subjects treated,—namely, the anatomy, physiology, and pathology of the teeth.

The second edition, while containing all that appeared in the first edition, has much that is new both in text and in illustration. In order to give an idea of the scope of the work a brief review of the subjects treated is appended:

*Anatomical Division.*—(1) The Oral Cavity; (2) The Superior Maxillary Bone; (3) The Inferior Maxillary Bone; (4) The Joint of the Inferior Maxillary Bone.

Under the general heading of The Teeth is to be found a description of the teeth in their general characteristics and a minute description of each tooth, both of the temporary and permanent set. Then follows a minute description of tooth dentine, enamel, cement, the dental pulp, and the gingiva. A good deal of space is devoted to the development of the teeth and the jaws, and histological considerations are naturally prominent. Much new material is here introduced, both in form of text and of plates.

The anatomical division is concluded by a chapter on the growth of the jaws.

The *Pathological Division* is opened by a consideration of Anomalies in Tooth Formation, and the subdivisions are, (1) Anomalies in Size; (2) Anomalies in Number; (3) Anomalies in Position; (4) Anomalies of Construction.

---

[1] Carl Wedl's Pathologie der Zähne. Zweite umgearbeitete Auflage. Herausgegeben von Dr. med. Univ. Joseph Ritter von Metnitz und Dr. med. Univ. Gustav Ritter von Wunschheim. Band I. Verlag von Arthur Felix, Leipzig.

The final chapter is devoted to misformations of the teeth or odontomes.

The first book of the second edition covers about one-third of the complete work and contains about thirty-five pages of new material. The new material adds much to the completeness of the work. It consists, in part, of quotations from leading German and Austrian dental periodicals; in part, of quotations from standard books, as those written upon oral subjects by Professor Zuckerkandl, of the University of Vienna; and in part, in the citation of cases which have appeared in the medical clinics of the University of Vienna. In addition, the editors have been free to add new material of their own, and to rearrange the old material. A successful attempt has been made to freshen up and bring down to date a treatise which, even in its original edition, is to-day a book of great value as a text- and reference-book.

As one example of the orderly and thorough arrangement of the material, I will quote from the section describing the influence of hereditary syphilis upon the teeth.

"Fournier draws attention to the following peculiarities (of syphilitic teeth):

"1. The tooth notch is almost always ground away obliquely at the expense of its forward border.

"2. The tooth has rounded corners.

"3. The vertical length of the tooth is shortened.

"4. The tooth is at times very narrow.

"5. The tooth has the form of a screw-driver; that is, it is thick and broad at its neck and narrowed at its free end.

"6. The long axes of the upper central incisors often converge.

"A further important consideration is that the Hutchinson tooth from youth on changes its form and entirely loses the characteristic notch. At twenty-five years there remains only a slight trace of the lesion in the form of a narrow oblique surface at the forward side of the cutting border. Over thirty years of age there are no longer Hutchinson teeth."

It is to be hoped that in due time this edition will be translated into English, so as to make it available for those who do not read the original.—WILLIAM H. POTTER, D.M.D., Boston.

